# Intracerebroventricular Streptozotocin Injections as a Model of Alzheimer’s Disease: in Search of a Relevant Mechanism

**DOI:** 10.1007/s12035-015-9132-3

**Published:** 2015-03-07

**Authors:** Paweł Grieb

**Affiliations:** Department of Experimental Pharmacology, Mossakowski Medical Research Centre, Polish Academy of Sciences, Str. Pawinskiego 5, 02-106 Warsaw, Poland

**Keywords:** Intracerebroventricular streptozotocin injection, Non-transgenic Alzheimer’s disease model, Insulin producing cells, Insulin receptors, Brain

## Abstract

Streptozotocin (STZ), a glucosamine-nitrosourea compound derived from soil bacteria and originally developed as an anticancer agent, in 1963 has been found to induce diabetes in experimental animals. Since then, systemic application of STZ became the most frequently studied experimental model of insulin-dependent (type 1) diabetes. The compound is selectively toxic toward insulin-producing pancreatic beta cells, which is explained as the result of its cellular uptake by the low-affinity glucose transporter 2 (GLUT2) protein located in their cell membranes. STZ cytotoxicity is mainly due to DNA alkylation which results in cellular necrosis. Besides pancreatic beta cells, STZ applied systemically damages also other organs expressing GLUT2, such as kidney and liver, whereas brain is not affected directly because blood-brain barrier lacks this transporter protein. However, single or double intracerebroventricular (icv) STZ injection(s) chronically decrease cerebral glucose uptake and produce multiple other effects that resemble molecular, pathological, and behavioral features of Alzheimer’s disease (AD). Taking into consideration that glucose hypometabolism is an early and persistent sign of AD and that Alzheimer’s brains present features of impaired insulin signaling, icv STZ injections are exploited by some investigators as a non-transgenic model of this disease and used for preclinical testing of pharmacological therapies for AD. While it has been assumed that icv STZ produces cerebral glucose hypometabolism and other effects directly through desensitizing brain insulin receptors, the evidence for such mechanism is poor. On the other hand, early data on insulin immunoreactivity showed intense insulin expression in the rodent brain, and the possibility of local production of insulin in the mammalian brain has never been conclusively excluded. Also, there are GLUT2-expressing cells in the brain, in particular in the circumventricular organs and hypothalamus; some of these cells may be involved in glucose sensing. Thus, icv STZ may damage brain glucose insulin producing cells and/or brain glucose sensors. Mechanistic explanation of the mode of action of icv STZ, which is currently lacking, would provide a valuable contribution to the field of animal models of Alzheimer’s disease.

## Introduction

Laboratory animals, mostly rodents, are commonly used as models of human diseases. The concept of such models is based on a certain degree of “analogy” between pathological processes that occur during progression of a disease in patients and in animals being studied as their substitute [1]. Although a model should not be considered identical to what is being modeled, it shall include a convergent set of analogies between the “target” phenomenon and the system being studied, i.e., between a patient in whom the disease develops and an experimental animal in which the “analogous” pathological processes occur. Creation of an animal model of a human disease requires at least some general knowledge about the disease mechanism. Provided sufficient degree of analogy, animal models should prove useful in efforts to understand details of disease process and in translational research for selection of experimental therapies prior to initiation of clinical evaluation. However, if the knowledge on which the model is based is unreliable, exploiting it may lead to false conclusions. This, indeed, happens quite frequently. Experimental treatments shown to be effective in animal studies in many cases turn out to be ineffective, sometimes even harmful, in clinical trials.

The case of Alzheimer’s disease (AD) is no exception. During the first half of the 1990 decade, mutations of the three “Alzheimer’s genes” coding beta-amyloid precursor protein (β-APP) and presenilins (PS-1, PS-2) have been determined as causative in the early onset autosomal dominant forms of this disease (familial AD) [2]. Early onset AD comprises only some 5 % of Alzheimer’s cases; notwithstanding the above, discovery of the Alzheimer’s genes paved the way to transgenic models of the disease. The first report on transgenic mice that express high levels of human mutated β-APP and progressively develop major pathological hallmarks of AD, i.e., extracellular amyloid beta deposits, neuritic plaques, and synaptic loss, was published in 1995 [3]. Since then, preclinical (translational) AD research has been dominated by a few murine transgenic models which simulate this neuropathology and display behavioral symptoms reminiscent of AD [4]. Hundreds of candidate drugs were tested with the use of these models, and more than 300 were found efficacious in ameliorating cognitive deficits, neuropathological burden of the model disease, or both. Unfortunately, none was translated into a successful therapy of human patients [5, 6]. Several factors were proposed as possible root causes of this failure. One of the most important may be the lack of robust approaches for identifying causality between multitude of interacting processes involved in the development of the disease [7]. Indeed, the issue of causation may be critical to the understanding pathophysiological mechanisms involved in Alzheimer’s disease.

While transgenic models are still the mainstream of AD research, alternative models are sought, which could prove more useful for translational research. The expert advisory panel gathered under the auspice of the Alzheimer’s Drug Discovery Foundation in the report published in 2011 [5] concluded that non-transgenic AD models were often underused. Recently, however, the model employing intracerebroventricular injection of a diabetogenic toxin streptozotocin (icv STZ model) is gaining considerable popularity. The aim of the present paper is to compare the assumptions underlying the icv STZ model and the transgenic models of AD and to discuss mechanism(s) which may be involved in the development of Alzheimer’s-like neuropathology in rodents following single or double injection of streptozotocin into the lateral ventricles. As will be shown, the major conceptual difference between the transgenic models and the icv STZ model concerns the place of brain glucose hypometabolism in the chain of causation leading to the Alzheimer’s neuropathology.

## Brain Glucose Hypometabolism in Alzheimer’s Disease: Secondary Effect or Primary Cause?

**Fig. 1:**
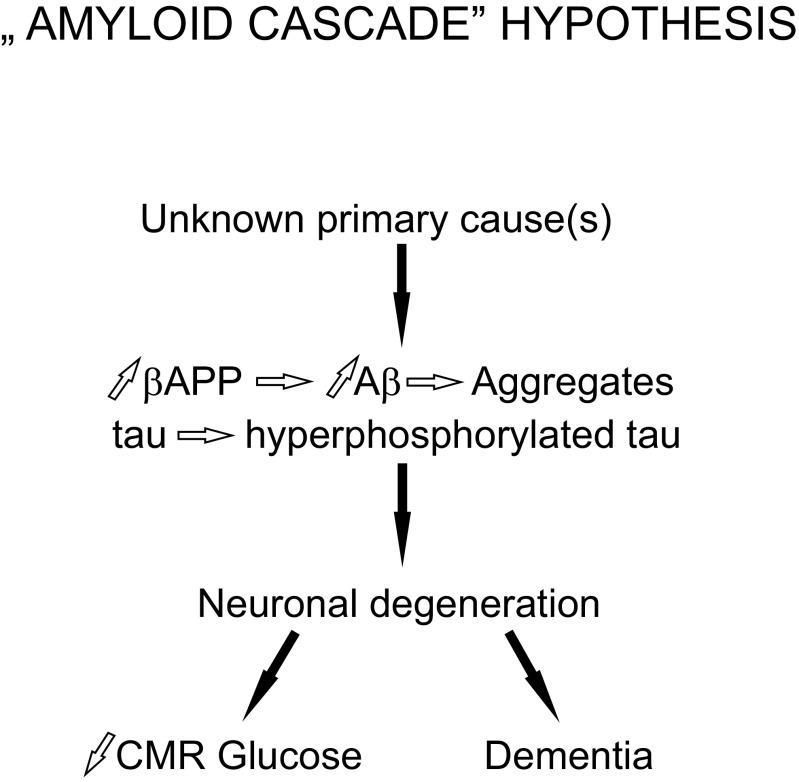
Diagram presenting chain of causation assumed by the “amyloid cascade” hypothesis

**Fig. 2:**
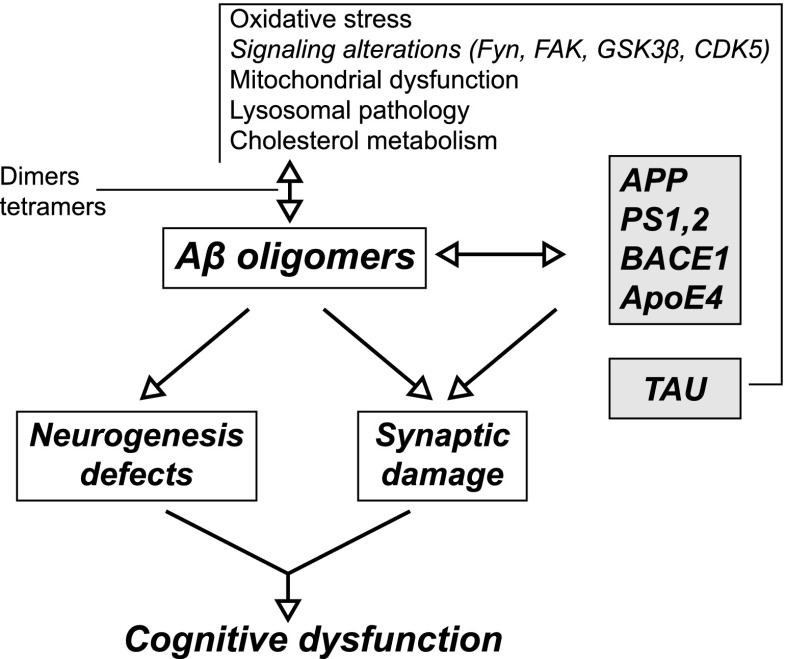
Diagram listing factors contributing to Aβ oligomerization and its downstream effects. Reproduced from ref. 11 (open access)

After the discovery of the Alzheimer’s genes, the leading concept underlying research in the AD area has been the “amyloid cascade” hypothesis proposed more than 20 years ago [8]. Its original version had two tenets, namely (i) that intracerebral deposition of β-amyloid (Aβ) is causative for AD pathology and (ii) that other brain lesions such as neurofibrillary tangles, dystrophic neurites, loss of synapses etc. follow. Since then, the hypothesis has been subject to several modifications to accommodate novel findings. For example, it is now assumed that neurotoxicity is exerted not by amyloid plaques located extracellularly but rather by intracellular fibrillary oligomeric forms of Aβ peptide formed earlier in the course of the disease [9]. However, the main tenet that the disease starts by Aβ accumulation, oligomerization, and toxicity remains unchanged. Although there must be some primary cause or trigger, such as genetic factors or unknown environmental toxicities, execution of neurodegeneration in AD starts at the level of Aβ oligomers and fibrils. The chain of causation in AD may thus be presented as shown in Fig. 1. Glucose hypometabolism in AD-prone brain regions such as hippocampus, although characteristic for AD even at its very early phase [10], is considered either the endpoint, or in any case the secondary effect of the neurodegenerative process. Even when not presented explicitly, such picture mirrors implicit understanding of the disease process. For example, in a typical review paper [11], numerous factors, metabolic and genetic, are listed as the causes or promoters of Aβ oligomerization leading to deficits of neurogenesis and synaptic damage, but decreased glucose consumption is not included (Fig. 2). In several other recent reviews which discuss possible causes of AD, brain metabolism of glucose is also not touched [12–15].

Decreasing brain glucose consumption is, however, a very early sign of AD. Measurements with the ^18^FDG-PET technique in individuals harboring Alzheimer’s genes have shown that signs of depressed brain glucose uptake, apparently independent of structural atrophy, are detectable up to three decades prior to the expected onset of clinical symptoms of early onset familial AD [16]. Moreover, there are other tracks of connection or relation between failure of brain glucose metabolism and Alzheimer’s disease. Incipient AD depression of brain glucose consumption is significantly more pronounced than depression of brain oxygen consumption. This disproportionality, abnormally high ratio of oxygen metabolic uptake to glucose metabolic uptake, has been found in the whole brain [17, 18], and imaging of brain oxygen and glucose consumption with positron emission tomography of oxygen-15 and 18-fluorodeoxyglucose revealed that it originates mostly in the parietotemporal region of the cerebral cortex [19].

Another track comes from in vitro and in vivo experiments in which brain glucose metabolism was blocked by metabolic inhibitors such as 2-deoxyglucose (2-DG). In vitro experiments on cells transfected with β-APP_695_ expresser plasmid have demonstrated that β-APP processing to potentially amyloidogenic COOH-terminal derivatives occurs in the endoplasmic reticulum or Golgi complex and is stimulated by exposing cells to a combination of 2-DG and sodium azide which inhibits cellular energy metabolism [20]. We [21] injected rats intraperitoneally with 500 mg/kg 2-DG and found that in the cerebral cortex grey matter, the level of β-APP is increased concomitantly with increase in the level of phosphorylated and decrease in dephosphorylated tau protein. Similar changes were later found by the others [22] following injection of single doses of 2-DG (1 g/kg), insulin, 3-nitropropionic acid, or kainic acid to mice, both wild-type and β-APP transgenic. Increase in brain β-APP was accompanied by increase in Aβ and β-secretase; these effects were attributed to pharmacological inhibition of energy production in the brain. However, in our experiments, 2-DG injection did not result in decreased cortical ATP or phosphocreatine, as judged from ^31^P-MRS spectra [21], which result was in agreement with the earlier report showing no fall in brain ATP assayed enzymatically following a single i.p. dose of 3 g/kg 2-DG in mice [23]. Proamyloidogenic effects of 2-DG may, therefore, result not from failing brain energetics coupled to mitochondrial oxidative metabolism but from decreased availability of glucose for other metabolic pathways, e.g., those involved in aerobic glycolysis.

In this context, it may be worth to mention that correlations have been found between deposition of amyloid plaques and the set of frontal and parietal midline structures with high connectivity and high metabolic activity (see e.g., [24] and the references quoted there). For example, Vlassenko et al. [25] analyzed the data obtained with three PET modalities mapping brain oxygen consumption (with ^15^O_2_), glucose consumption (with ^19^ F-deoxyglucose) and amyloid β burden (with ^13^C-PIB) and found that the spatial distribution of aerobic glycolysis in normal young adults correlated with the location of Aβ deposits in AD patients as well, as in cognitively normal participants with elevated Aβ. The authors suggested a possible link between regional aerobic glycolysis in young adulthood and later development of Alzheimer pathology.

**Fig. 3:**
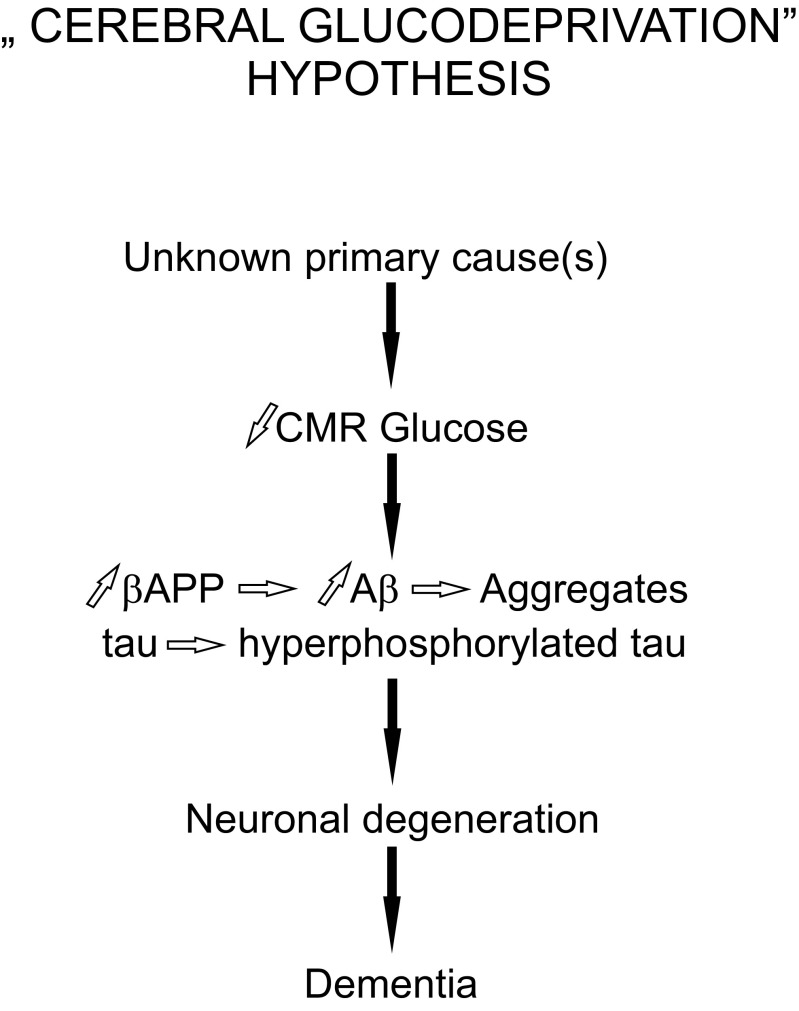
Diagram presenting chain of causation assumed by the “cerebral glucodeprivation” hypothesis

Considering the above, an alternative chain of causation may be proposed for AD (Fig. 3), in which decreased ability of brain tissues to metabolize glucose is the central point at which execution of neurodegeneration is initiated, whereas development of amyloid deposits and neurofibrillary tangles built of hyperphosphorylated tau protein follow. In an analogy to type 2 diabetes, the hallmark of which is decreased ability of peripheral tissues to metabolize glucose, brain-specific decrease of the ability to metabolize glucose could be called type 3 diabetes, and indeed, such term has been proposed for Alzheimer’s disease [26].

## Streptozotocin-Based Model of Diabetes

**Fig. 4:**
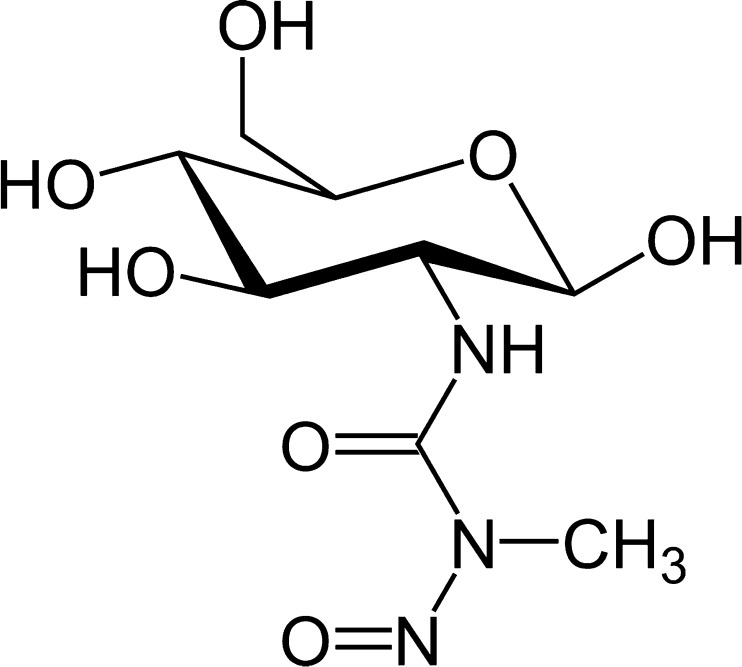
Structural formula of streptozotocin (STZ)

Discussion on the streptozotocin intracerebroventricular injections (icv STZ), experimental paradigm as a model of Alzheimer’s disease will be preceeded with a brief summary of the history and properties of this substance. Streptozotocin (structural formula, Fig. 4), isolated in the late 1950s from a strain of the soil bacteria *Streptomyces achromogenes*, was patented and initially developed as an antibiotic, later as an anticancer agent. Until now, it is marginally used in the multi-drug therapy of some rare neuroendocrine tumors [27], although is much more popular in the non-clinical research, because injected intravenously or intraperitoneally induces diabetes in experimental animals.

More than 40 years ago, it has been shown that in the rat (Wistar strain), single intravenous injection of STZ results in dose-dependent diabetogenic response consisting of increase in serum glucose level and urine volume and decrease in serum and pancreatic immunoreactive insulin; doses of 45–65 mg/kg decreased insulin by more than 10 times [28]. Currently, it is accepted that intravenous or intraperitoneal injection of STZ in dose of 40–60 mg/kg or higher is the model of type 1 (insulin-dependent) diabetes [29, 30]. To obtain a model of type 2 diabetes (non-insulin-dependent) characterized by impaired glucose-stimulated insulin secretion and insulin resistance, i.e., inadequate response to insulin signal in muscles and other tissues, lower peripheral STZ dose (35 mg/kg or less) is combined with high fat diet (see e.g., [31]).

Current understanding of the diabetogenic property of streptozotocin and mechanisms involved has been summarized by Lenzen [32, 33]. Unlike other nitrosureas which are lipophilic and quickly taken up by cells, STZ due to hexose substitution is less lipophilic and requires a particular glucose transporter, GLUT2, to cross cellular membranes. Selectivity of STZ toward insulin producing beta cells in pancreas is explained by the presence of GLUT2 in the membranes of vulnerable cells. Acting intracellularly methylnitrosourea moiety of STZ alkylates DNA; cell death, which occurs by necrosis, is the consequence of poly(ADP-ribose)polymerase (PARP) activation with subsequent depletion of NAD^+^ and ultimately depletion of ATP stores. Besides insulin-producing pancreatic beta cells, STZ is toxic toward other organs expressing GLUT2 transporter, particularly kidney and liver. Brain is not affected directly because STZ is not able to penetrate blood-brain barrier which lacks GLUT2 transporter.

Streptozotocin action is considered similar to that of the other well-known diabetogenic substance alloxan. Both compounds are taken up by GLUT2 transporters, act intracellularly, are selectively toxic toward pancreatic insulin-producing β cells, and their toxicity can be blocked by peripheral application of non-metabolysable glucose analog 3-*O*-methyl-glucose. However, mechanisms of their diabetogenic activity seem to differ in some points. One difference is that D-glucose and D-mannose are reportedly able to block toxic effect of alloxan but not STZ [29]. The other concerns chemical stability: At pH 7.4 and 37 °C, alloxan is very unstable with half-life of 1.5 min; STZ is thought to be relatively stable for at least 1 h [33], although the other source indicates its half-life in solution at physiological pH as 19 min [34].

Cytotoxicity of STZ is dependent on the activity of poly (ADP-ribose) polymerase (PARP), and PARP-knockout mice are resistant to this toxin [35, 36]. Currently accepted view is that PARP activation in various pathophysiological situations is the consequence of increased tissue ROS generation [37]. In the long term, animals made chronically hyperglycemic by STZ develop oxidative stress because hyperglycemia increases generation of free radicals [38]). Likewise, although diabetes induced in rodents by systemic STZ injection affects several aspects of central nervous system metabolism and function (see, for example [39, 40]), it is implicitly assumed that all central nervous system effects of STZ given peripherally are indirect, i.e., can be attributed to chronic hyperglycemia and its sequelae such as tissue oxidative stress, etc.

GLUT2 glucose transporting protein is a part of the distributed glucose sensing mechanism which is located in the pancreatic beta cells and some other organs, e.g., liver [41]. GLUT2 is also expressed in the brain, in particular in hypothalamus and brainstem, where it is encountered in neurons, astrocytes, tanycytes, and epithelial cells lining the cerebral ventricles (see references cited in [42]). Of these cells, hypothalamic glucose-sensing neurons are located inside the blood-brain barrier. However tanycytes, a particular type of glial cells located in the circumventricular organs devoid of blood-brain barrier [43], are suspected to be able to sense blood glucose level in both cerebrospinal fluid and blood [44, 45].

## Intracerebroventricular Administration of Streptozotocin and Alloxan: Similarities and Differences

Intracerebroventricular instillations of drugs are rarely used in the clinic for administering drugs [46]; more frequently, drugs or selective toxicants are applied to the brain ventricular system of laboratory animals for experimental purposes. Injections are mostly performed into the lateral ventricles. Although, as pointed out by Pardridge [47], drug injected into the cerebrospinal fluid will rapidly move into the blood via bulk flow but will poorly penetrate into the brain tissue owing to limitations of diffusion, many investigators assume that intracerebroventricular (icv) administration allows high concentrations to enter the central compartment because it bypasses the blood-brain barrier and other mechanisms that limit distribution of the administered substance into the brain.

More than 30 years ago, icv injections of alloxan were used, apparently for the first time, in experiments aimed at elucidating intracerebral glucose sensing and its physiological role [48]. Interestingly, Ritter et al. [49] reported soon after that alloxan injections into the lateral ventricles (dose 40 μg in 5 mL) result in marked reduction of glucoprivic feeding evoked by subcutaneous 2-deoxyglucose or insulin, whereas icv streptozotocin in doses up to 400 μg were ineffective. No explanation for the different reaction to alloxan and streptozotocin has been provided, but in further studies of cerebral glucosensing, neurons icv alloxan injections have been preferred [50]. Currently, it is believed that cerebral glucose sensing is involved in the control of feeding behavior, peripheral glucose homeostasis, and thermoregulation. Its cellular mechanisms are far from being understood, but by analogy to pancreatic β cells, it is supposed by some investigators that they involve GLUT2 glucose transporter [51].

Lacković and Salković [52] compared the effects of peripheral and intracerebroventricular alloxan and streptozotocin on rat brain monoamines. Eight days after subcutaneous administration of the diabetogenic doses of alloxan increased brain serotonin, dopamine and noradrenaline were noted, but no such effect occurred after intraperitoneal injections of diabetogenic doses of streptozotocin. However, the effects of icv alloxan and STZ were similar. On the other hand, further research has revealed that icv administration of alloxan does not alter rat memory and learning functions assessed with behavioral tests 3 months later [53], whereas icv STZ resulted in cognitive deficits which became evident 2 weeks after the treatment and persisted at least up to 3 weeks post-treatment [54]. Likely because of this difference, the cause of which also remains unexplained, icv streptozotocin but not icv alloxan has been developed as a non-transgenic model of Alzheimer’s disease.

## Intracerebroventricular Streptozotocin: a Metabolic Model of Alzheimer’s Disease

The pioneer of the icv STZ model is Professor Sigfried Hoyer. His early works on this subject have been summarized in the paper published in 1994 [55]. Current status of the concept that icv STZ is the non-transgenic metabolic model of sporadic AD has been presented in the review with his coauthorship, published in January 2013 [56].

The starting point of the reasoning developed by Hoyer was that in the sporadic Alzheimer’s disease, there is a fall in brain consumption of both oxygen and glucose, but a fall in brain oxygen consumption is disproportionately slighter. These observations led to the hypothesis that the major biochemical perturbation in incipient sporadic AD concerns the control over cerebral glucose metabolism, which is subsequent to a signal transduction failure of the cerebral insulin receptor. At this point, another observation was also taken into account, namely that in adult male Wistar rats, intracerebroventricular administration of 80 mU insulin stimulated two key glycolytic enzymes, hexokinase and phosphofructokinase, by approx. 20 %. This later result was interpreted as the evidence for the control of glycolytic flux in the brain by insulin and led to the conclusion that any perturbation in insulin signal transduction will have severe impact on brain glucose metabolism [57]. The next step was to administer streptozotocin intraventricularly, in order to damage the control level of cerebral glucose/energy metabolism. In the other paper from the Hoyer’s group [58], the purpose of the study was defined as to investigate whether or not cerebral glucose utilization is changed locally after intracerebroventricular (icv) STZ administered in a subdiabetogenic dosage (1.5 mg/kg bw.). These and other similar experiments have shown that application of STZ to the rat lateral ventricles decreased brain glucose utilization, in particular in the frontal and parietal cortex, decreased ATP and phosphocreatine concentrations, ATP/ADP ratio, and the energy charge potential in the cerebral cortex [59], and produced several other brain metabolic and behavioral disturbances. In one paper [60], it was hypothesized that triple icv STZ injections inhibit cerebral insulin receptors, but no mechanistic explanation was provided for such effect.

Based on the close analogy between human patients with AD and rats subjected to icv STZ injections in respect to brain metabolic and behavioral disturbances, Hoyer proposed that icv STZ shall be recognized as the non-transgenic preclinical model of Alzheimer’s disease. In the most recent review [56], this opinion is further extended by the statement that sporadic AD as well as its model obtained by icv injection of STZ are being recognized as an insulin resistant brain state (IRBS). However, the exact nature of IRBS is only vaguely defined. The preceding review dedicated to the hypothetical concept of IRBS as the cause of Alzheimer disease [61] explains that functionally, the IRBS may correspond to the pathophysiology of diabetes mellitus type II. However, in the case of sporadic AD, the authors propose the term IRBS instead of “diabetes mellitus type II confined to the brain” to avoid misunderstandings. Recent reviews dedicated to the IRBS concept written by the others [62, 63] also do not include rigorous definition of this term. One may guess that IRBS is a brain analog of insulin resistance syndrome (IRS) of peripheral tissues, a condition characterized by decreased tissue sensitivity to the action of insulin and leading to a compensatory increase in insulin secretion [64]. It shall be noted, however, that in the (peripheral) IRS, chronic hyperisulinemia in blood occurs concomitantly with reduced insulin levels in the central nervous system, explained its by reduced delivery from pancreatic source [65].

Development of several Alzheimer-like indices of brain metabolic and behavioral disturbances in rats subjected to one or two icv STZ injection(s) is considered to be a dynamic process. In the majority of papers, the effects of icv STZ on brain function and behavior were followed within a relatively short time of a few weeks. Only recently, the Hoyer’s group reported on the longer follow-up of icv STZ rats which revealed deposits of amyloid β in the wall of meningeal and cortical blood vessels visualized by thioflavine-S and Congo red as well, as by immunohistochemical staining [66]. In one of the most recent reviews [56], it is stated that cognitive and neurochemical changes triggered by icv STZ injection(s) follow a time-dependent pattern which consists of three phases. Within 1 month, an acute response develops, at 1 to 3 months, a tendency to return to normal values takes place and finally, at 6 to 9 months, decompensation phase occurs with a slow and progressive aggravation. This temporal evolution of pathology initiated by (single or double) streptozotocin injection into the lateral ventricles seems to resemble the situation in a real life which does not exist in the transgenic mice AD models.

**Fig. 5:**
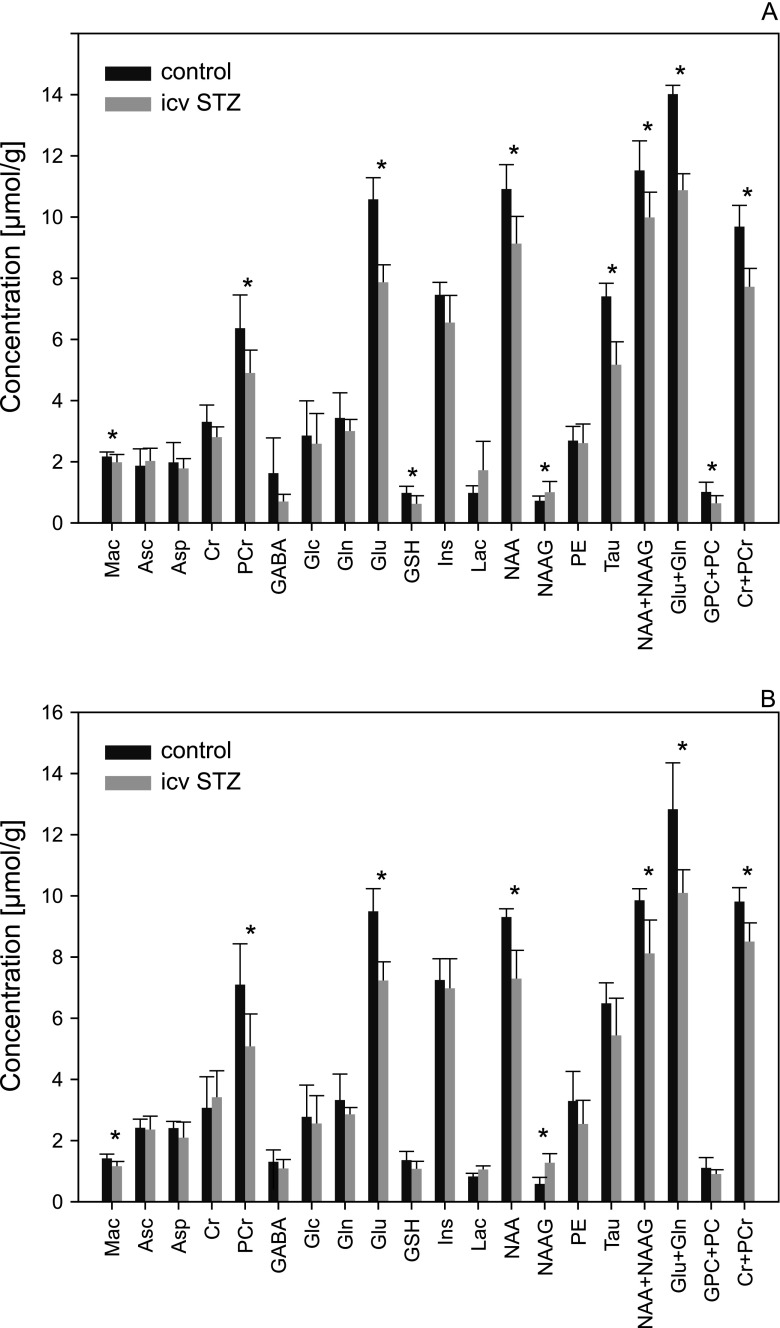
Effects of icv STZ (bilaterally, 3 mg/kg body weight, on days 1 and 3) on concentrations of brain metabolites obtained from ^1^H-MRS spectra recorded from 27 μL volume comprising hippocampus and part of the cerebral cortex, in 7 T magnetic field strength with very short echo time. **a** 2 weeks after and **b** 2 months after icv STZ injection. *Mac* macromolecules, *Asc* ascorbate, *Asp* aspartate, *Cr* creatine, *PCr* phosphocreatine, *Glc* glucose, *Gln* glutamine, *Glu* glutamate, *Ins* inositol, *Lac* lactate, *NAA N*-acetylaspartate, *NAAG N*-acetylaspartylglutamate, *PE* phosphorylethanolamine, *Tau* taurine; *GPC+PC* glycerophosphocholine and phosphocholine, *n* = 8, *error bars* show standard deviations, *asterisks* indicate *p* < 0.05 by two-tailed *t* test for independent means. (Reproduced from ref. 68, with permission from Springer-Verlag)

The other authors also reported on various neurochemical and neuropathological consequences of icv STZ injection(s). Most observations were convergent with the Hoyer’s observations that metabolic disturbances following icv STZ cover large areas of the brain, including locations distant from the injection site, and that they mount up in time. For example, two daily injections of STZ into the lateral ventricles of adult rats caused in the frontoparietal cortex a significant enlargement of the neuronal Golgi apparatus detected 3 weeks later [67], and similar experimental paradigm led to indiscriminate lowering of most proton magnetic resonance lines recorded from the 27 μL tissue volume which included hippocampus and a part of cerebral cortex [68] (Fig. 5). Five weeks after a single bilateral icv STZ injection (3 mg/kg), Coreia et al. [69] have found a decrease in brain weight, a cognitive decline, a significant increase in hippocampal Aβ (1–42), and increase in both hippocampal and cortical hyperphosphorylated tau levels. Another important features of rat icv STZ model which resemble findings in brains from AD patients are signs of brain tissue oxidative stress (see e.g., [70]) and brain mitochondrial abnormalities which may cause caspase-mediated apoptotic cell death [71, 72]. Mitochondrial abnormalities and tissue oxidative stress may be related, although according to a recent data [73] glial activation, TNF-α and free radical generation following icv STZ occurs earlier than apoptosis and synaptic neurotoxicity. In the other study [74], in rats after icv STZ, different types of behavioral and neurodegenerative responses have been observed with distinct time courses ranging from 1 h to 15 days.

In the brains of adult icv STZ-treated rats, Deng et al. [75] found impaired insulin signaling, overactivation of glycogen synthase kinase-3β, decreased levels of major brain glucose transporters, downregulated protein *O*-GlcNAcylation, as well as increased phosphorylation of tau and neurofilaments and decreased microtubule-binding activity of tau. These authors suggested that the key mechanism in the development of disease in the human AD, as well as in the rodent model disease is the development of brain insulin resistance which leads to neurofibrillary degeneration via two cooperating pathways, namely decreased PI3K-AKT signaling activity and glycogen synthase kinase 3 beta (GSK3β) overactivation and decreased GLUT1/3 expression and decreased intraneuronal glucose metabolism. More recently, investigators from the same laboratory [72] compared the non-cognitive and cognitive behaviors as well as biochemical and immunohistochemical alterations in brains of a transgenic and a non-transgenic murine model of AD, 3xTg-AD mice which harbor mutated presenilin 1, APP, and tau genes and icv STZ mice, respectively. The conclusion was that both models demonstrated similar behavioral patterns, whereas they differed in some neuropathological features; the most prominent brain abnormality in the icv STZ model was neuroinflammation, whereas in the transgenic model, it was elevation of hyperphosphorylated tau. Finally, Chen et al. [76] observed that icv STZ injection exacerbates memory disturbances and Alzheimer-like neurochemical changes in brains of 3xTg-AD mice, concluding that these findings demonstrate the role of metabolic insult in AD pathology in vivo.

Recently, de la Monte [77] and de la Monte and Tong [78] pursued the concept of Alzheimer’s disease being type 3 (brain-specific) diabetes. Whereas this idea stemmed from clinical observations suggestive of that AD is a metabolic disease in which brain cannot efficiently utilize glucose, experimental observations of impairments of insulin, as well as insulin growth factors (IGF-1, IGF-2) signaling following icv STZ in rat pups [79, 80] were interpreted as providing the strongest evidence favoring this concept. Interestingly, in these experiments, a significant atrophy of the whole brain was observed 3 weeks after icv STZ injection [79].

However, the results obtained by some other authors are suggestive of spatial restriction of intracerebral damage caused by STZ injection into lateral ventricles. For example, Terwel et al. [81] injected adult rats intracerebroventricularly with STZ and 3 weeks later investigated activities of chosen enzymes in two brain areas and found that in the septum activities of all enzymes except choline acetyltransferase (ChAT) were decreased, whereas in the hippocampus the decrease was limited to ChAT. Moreover, in icv STZ-injected animals, the septum weight was reduced whereas the hippocampus weight was unchanged. These findings were interpreted as suggestive of that STZ injected into the lateral ventricles acts as a non-selective neurotoxin near the site of injection; sparing septal ChAT activity could be explained by the more distant location of the septal cholinergic neurons to the injection site. Shoham et al. [82] provided histopathological evidence that damage caused by icv STZ is restricted to axons and myelin of the fornix, anterior hippocampus and periventricular structures known to be essential for learning and spatial memory. They concluded that learning deficits caused by icv STZ may not be caused only by impairing brain glucose utilization. More recently, Kraska et al. [83] reported that in rats, the extent and dynamics of brain neurodegeneration following icv STZ injection depends on the amount of the toxin injected. Higher dose (1 mg/kg) induced severe and acute neurodegenerative lesions in the septum and corpus callosum, which were associated with inflammation and might have been caused by local oxidative stress, whereas lower dose led to less severe but more chronic and widespread effects.

## Does icv STZ Cause Brain Insulin Receptors Desensitization Directly? Are Other Explanations Plausible?

It is frequently assumed that streptozotocin injected intraventricularly damages or desensitizes brain insulin receptors or otherwise produces brain insulin resistance defined as inadequate response of insulin receptors located in the brain to insulin. This interpretation is sometimes presented as an analogy between the mechanism of action of icv STZ on the brain and the mechanism of peripheral action of low doses of STZ. For example, in the (already brought up several times) authoritative review on the icv STZ model of AD, Salkovic-Petrisic et al. [56] opined that multiple parenteral treatment with low to moderate STZ doses causes insulin resistance by damaging insulin receptor (IR) signaling. The problem with this statement is that STZ given peripherally does not damage insulin receptor signaling directly. Three references quoted there to support such opinion [84–86] describe experiments in which rats were made severely diabetic by injecting peripherally high streptozotocin doses: 45, 65, and 100 mg/kg, respectively. Moreover, all these experiments showed that development of in vivo insulin resistance in peripheral tissues following subcutaneous or intravenous STZ injection is secondary to insulin deficiency in blood and hyperglycemia. For example, in the abstract of their paper, Blondel and Portha [84] wrote that insulin deficiency and concomitant hyperglycemia, as the consequences of streptozotocin administration in the adult rat, rapidly lead to the development of in vivo insulin resistance, first in the liver and later on in the peripheral tissues.

It shall also be taken into account that insulin receptors, at least in some extracranial locations, are subject to intracellular turnover, being continuously synthesized, deposited in cell membranes, endocytosed, and degraded with a relatively short half-life of hours rather than days (for example, 6.7 h in differentiating adipocytes [87]). If similar IR turnover operates in the brain, inflicting IR damage but leaving cells bearing these receptors intact would result in their rather quick regeneration. Last but not least, STZ molecule is structurally very different from insulin molecule, and there is no apparent reason to expect interaction between STZ and IR. It seems that the hypothesis of IR damage throughout the brain caused directly by single or double intracerebroventricular injection of STZ cannot explain the apparent irreversibility and long-lasting progression of the evoked effects.

Of note is also that brain neuron-specific insulin receptor knockout (NIRKO) mice, which exhibit a complete loss of insulin-mediated activation of phosphatidylinositol 3-kinase, were reported to display markedly reduced phosphorylation of Akt and GSK3 beta kinases leading to substantially increased phosphorylation of Tau protein but exhibited no alteration in neuronal proliferation/survival, memory, or basal brain glucose metabolism [88].

The next relevant issue is that of the diffusion of streptozotocin after intracerebrovientricular injection. As pointed out by Pardridge [47], drug injected into the cerebrospinal fluid will rapidly move into the blood via bulk flow but will poorly penetrate into the brain tissue owing to limitations of diffusion. Data obtained recently with fluorescent tracers of differing molecular weights injected into the lateral ventricle of mice [89] indicate that substances injected into ventricular CSF only minimally enter brain parenchyma and remain confined to the immediate periventricular region. Therefore, it is not possible that single injection of STZ into the lateral ventricles evokes metabolic and cytoarchitectonic responses in such distant locations as frontal or parietal cortex through damaging insulin receptors which are present there. It may be concluded that the hypothesis that streptozotocin injected once or twice to the cerebral lateral ventricles damages insulin receptors throughout the brain acting directly on them is not a plausible explanation.

The other issue which deserves reflection in the context of icv STZ model of AD is the possibility of intracerebral synthesis of insulin in mammals. In a classical study of Havrankova et al. [90], no change in either brain insulin or brain insulin receptors was found within up to 30 days after injection of a diabetogenic peripheral STZ dose which led to depletion of insulin in pancreas and blood. Later, it was found that insulin concentrations in brain displayed no relationship to the serum or CSF insulin levels [91]. These observations were taken as the evidence for insulin synthesis in the brain. Most of the further research did not confirm such hypothesis, and many authors believe that all insulin found in mammalian brains comes from pancreas (see e.g., [92]). However, others are not certain (see e.g., [62]). Schechter et al. [93], using insulin knockout mice able to survive only 3 days after birth, provided evidence for a role of insulin within the brain in maintaining a balance in phosphorylation of neuronal cytoskeleton and pointed out that pancreatic insulin with its short half-life of a couple of minutes would be a difficult source to constantly maintain the cytoskeleton equilibrium.

Involvement of GLUT2 as mediator of icv STZ effects has been postulated [83, 94, 95]. GLUT2 is the isoform present in pancreatic insulin producing cells and also involved in glucose sensing. Within the body, there is a distributed system of glucose sensors, and part of this system is located in the brain [42]. GLUT2 protein immunoreactivity has been detected in many brain areas [96]. In particular, a subpopulation of ependymal cells called tanycytes, located in the lateral ventricles and other circumventricular organs, contain high density of GLUT2 protein, and studies have shown that these transporters are functional [97]. It may be expected that, due to their location close to the injection site, tanycytes are preferentially damaged by a single icv STZ injection. Some researchers postulated that these cells monitor glucose levels in both CSF and plasma. What happens if they are irreversibly damaged by a single or double icv STZ injection and signals which they deliver to the hypothalamic integrator vanish?

Almost 30 years ago, Coimbra and Migliorini [98] discovered that microinjections of minute amounts of 2-deoxyglucose into the preoptic area of fed rats induced rapid increases in blood plasma free fatty acids (FFAs), whereas glucose remained unchanged. Acute exposure to free fatty acids is known to cause insulin resistance in muscle [99], and FFA are able to cross blood-brain barrier [100]; moreover, they are implicated in the generation of the amyloid peptides in the brain [101, 102]. It is tempting to speculate that single or double icv STZ injection may chronically influence metabolism in brain regions distant from injection site indirectly, through changes in plasma level of FFA or other substances potentially toxic to the brain. Another mechanism by which changes in plasma content could possibly influence brain glucose metabolism may involve neurotoxic ceramides which may enter brain from blood through blood-brain barrier and induce brain insulin resistance, inflammation, and cell death [103]. Although involvement of the aforementioned mechanisms is currently not supported by any direct evidence, a possibility of some extracranial mechanisms contributing to the central effects of streptozotocin injected intracerebroventricularly should not be a priori excluded. Interestingly, although intraperitoneal and intracerebroventricular injection(s) of STZ are known to produce different effects on brain metabolism, in the case of another nitrosamino compound *N*-nitrosodiethylamine (NDEA), the effects were similar [104].

In summary, there is no doubt that single or double injection of streptozotocin into the lateral ventricles of rat produces chronic metabolic, neuropathological, and behavioral disturbances reminiscent of human Alzheimer’s disease, but acceptable mechanistic explanation for these phenomena is lacking. The concept of irreversible inhibition or desensitization of insulin receptors caused directly by STZ diffusing throughout the brain parenchyma does not seem plausible, and alternative explanation(s) shall be sought. Understanding causality between multitude of interacting processes involved in the development of the model disease evoked by icv STZ injection(s) is necessary for assessment of its convergence with the human sporadic Alzheimer’s disease and possible usefulness in translational research.

## References

[1] Hau J, Conn PM (2008). Animal models of human diseases. An overview. (in:) Sourcebook of Models for Biomedical Research.

[2] Bertram L, Tanzi RE (2012). The genetics of Alzheimer's disease. Prog Mol Biol Transl Sci.

[3] Games D, Adams D, Alessandrini R, Barbour R, Berthelette P, Blackwell C, Carr T, Clemens J (1995). Alzheimer-type neuropathology in transgenic mice overexpressing V717F beta-amyloid precursor protein. Nature.

[4] Li C, Ebrahimi A, Schluesener H (2013). Drug pipeline in neurodegeneration based on transgenic mice models of Alzheimer's disease. Ageing Res Rev.

[5] Shineman DW, Basi GS, Bizon JL, Colton CA, Greenberg BD, Hollister BA, Lincecum J, Leblanc GG (2011). Accelerating drug discovery for Alzheimer's disease: best practices for preclinical animal studies. Alzheimers Res Ther.

[6] Zahs KR, Ashe KH (2010). 'Too much good news'—are Alzheimer mouse models trying to tell us how to prevent, not cure, Alzheimer's disease?. Trends Neurosci.

[7] Geerts H (2009). Of mice and men: bridging the translational disconnect in CNS drug discovery. CNS Drugs.

[8] Hardy JA, Higgins GA (1992). Alzheimer's disease: the amyloid cascade hypothesis. Science.

[9] Gilbert BJ (2013). The role of amyloid β in the pathogenesis of Alzheimer's disease. J Clin Pathol.

[10] Mosconi L, De Santi S, Li J, Tsui WH, Li Y, Boppana M, Laska E, Rusinek H (2008). Hippocampal hypometabolism predicts cognitive decline from normal aging. Neurobiol Aging.

[11] Crews L, Rockenstein E, Masliah E (2010). APP transgenic modeling of Alzheimer's disease: mechanisms of neurodegeneration and aberrant neurogenesis. Brain Struct Funct.

[12] Armstrong RA (2013). What causes Alzheimer's disease?. Folia Neuropathol.

[13] Mondragón-Rodríguez S, Basurto-Islas G, Lee HG, Perry G, Zhu X, Castellani RJ, Smith MA (2010). Causes versus effects: the increasing complexities of Alzheimer's disease pathogenesis. Expert Rev Neurother.

[14] Skaper SD (2012). Alzheimer's disease and amyloid: culprit or coincidence?. Int Rev Neurobiol.

[15] Swerdlow RH (2012). Alzheimer's disease pathologic cascades: who comes first, what drives what. Neurotox Res.

[16] Mosconi L, Sorbi S, de Leon MJ, Li Y, Nacmias B, Myoung PS, Tsui W, Ginestroni A (2006). Hypometabolism exceeds atrophy in presymptomatic early-onset familial Alzheimer's disease. J Nucl Med.

[17] Hoyer S, Nitsch R, Oesterreich K (1991). Predominant abnormality in cerebral glucose utilization in late-onset dementia of the Alzheimer type: a cross-sectional comparison against advanced late-onset and incipient early-onset cases. J Neural Transm Park Dis Dement Sect.

[18] Ogawa M, Fukuyama H, Ouchi Y, Yamauchi H, Kimura J (1996). Altered energy metabolism in Alzheimer's disease. J Neurol Sci.

[19] Fukuyama H, Ogawa M, Yamauchi H, Yamaguchi S, Kimura J, Yonekura Y, Konishi J (1994). Altered cerebral energy metabolism in Alzheimer's disease: a PET study. J Nucl Med.

[20] Gabuzda D, Busciglio J, Chen L, Matsudaira P, Yankner B (1994). Inhibition of energy metabolism alters the processing of amyloid precursor protein and induces a potentially amyloidogenic derivative. J Biol Chem.

[21] Grieb P, Gordon-Krajcer W, Frontczak-Baniewicz M, Walski M, Ryba MS, Kryczka T, Fiedorowicz M, Kulinowski P (2004). 2-deoxyglucose induces beta-APP overexpression, tau hyperphosphorylation and expansion of the trans-part of the Golgi complex in rat cerebral cortex. Acta Neurobiol Exp (Wars).

[22] Velliquette RA, O'Connor T, Vassar R (2005). Energy inhibition elevates beta-secretase levels and activity and is potentially amyloidogenic in APP transgenic mice: possible early events in Alzheimer's disease pathogenesis. J Neurosci.

[23] Horton RW, Meldrum BS, Bachelard HS (1973). Enzymic and cerebral metabolic effects of 2-deoxy-D-glucose. J Neurochem.

[24] Myers N, Pasquini L, Göttler J, Grimmer T, Koch K, Ortner M, Neitzel J, Mühlau M (2014). Within-patient correspondence of amyloid-β and intrinsic network connectivity in Alzheimer's disease. Brain.

[25] Vlassenko AG, Vaishnavi SN, Couture L, Sacco D, Shannon BJ, Mach RH, Morris JC, Raichle ME (2010). Spatial correlation between brain aerobic glycolysis and amyloid-β (Aβ) deposition. Proc Natl Acad Sci U S A.

[26] Steen E, Terry BM, Rivera EJ, Cannon JL, Neely TR, Tavares R, Xu XJ, Wands JR (2005). Impaired insulin and insulin-like growth factor expression and signaling mechanisms in Alzheimer’s disease—is this type 3 diabetes?. J Alzheimers Dis.

[27] Turner NC, Strauss SJ, Sarker D, Gillmore R, Kirkwood A, Hackshaw A, Papadopoulou A, Bell J (2010). Chemotherapy with 5-fluorouracil, cisplatin and streptozocin for neuroendocrine tumours. Br J Cancer.

[28] Junod A, Lambert AE, Stauffacher W, Renold AE (1969). Diabetogenic action of streptozotocin: relationship of dose to metabolic response. J Clin Invest.

[29] Ganda OP, Rossini AA, Like AA (1976). Studies on streptozotocin diabetes. Diabetes.

[30] Degenhardt TP, Alderson NL, Arrington DD, Beattie RJ, Basgen JM, Steffes MW, Thorpe SR, Baynes JW (2002). Pyridoxamine inhibits early renal disease and dyslipidemia in the streptozotocin-diabetic rat. Kidney Int.

[31] Srinivasan K, Viswanad B, Asrat L, Kaul CL, Ramarao P (2005). Combination of high-fat diet-fed and low-dose streptozotocin-treated rat: a model for type 2 diabetes and pharmacological screening. Pharmacol Res.

[32] Lenzen S (2007) Alloxan and streptozotocin diabetes. In: Peschke E (ed) Endokrinologie III Vorträge im Rahmen des Projektes ‘Zeitstrukturen endokriner Systeme’. [Endocrinology III lectures within the ‘time structures of endocrine systems’ project framework]. Abhandlung der Sächs. Akad. Wiss., Mathnaturwiss Klasse, Verlag der Sächsischen Akademie der Wissenschaften, Leipzig, commissioned by S. Hirzel Verlag, Stuttgart/Leipzig, pp 119–138

[33] Lenzen S (2008). The mechanisms of alloxan- and streptozotocin-induced diabetes. Diabetologia.

[34] Müller D, Nitsch RM, Wurtman RJ, Hoyer S (1998). Streptozotocin increases free fatty acids and decreases phospholipids in rat brain. J Neural Transm.

[35] Masutani M, Suzuki H, Kamada N, Watanabe M, Ueda O, Nozaki T, Jishage K, Watanabe T (1999). Poly(ADP-ribose) polymerase gene disruption conferred mice resistant to streptozotocin-induced diabetes. Proc Natl Acad Sci U S A.

[36] Pieper AA, Brat DJ, Krug DK, Watkins CC, Gupta A, Blackshaw S, Verma A, Wang ZQ (1999). Poly(ADP-ribose) polymerase-deficient mice are protected from streptozotocin-induced diabetes. Proc Natl Acad Sci U S A.

[37] Cuzzocrea S (2005). Shock, inflammation and PARP. Pharmacol Res.

[38] King GL, Loeken MR (2004). Hyperglycemia-induced oxidative stress in diabetic complications. Histochem Cell Biol.

[39] Artola A, Kamal A, Ramakers GM, Biessels GJ, Gispen WH (2005). Diabetes mellitus concomitantly facilitates the induction of long-term depression and inhibits that of long-term potentiation in hippocampus. Eur J Neurosci.

[40] Beauquis J, Roig P, Homo-Delarche F, De Nicola A, Saravia F (2006). Reduced hippocampal neurogenesis and number of hilar neurones in streptozotocin-induced diabetic mice: reversion by antidepressant treatment. Eur J Neurosci.

[41] Seyer P, Vallois D, Poitry-Yamate C, Schütz F, Metref S, Tarussio D, Maechler P, Staels B (2013). Hepatic glucose sensing is required to preserve β cell glucose competence. J Clin Invest.

[42] Thorens B (2011). Brain glucose sensing and neural regulation of insulin and glucagon secretion. Diabetes Obes Metab.

[43] Langlet F, Mullier A, Bouret SG, Prevot V, Dehouck B (2013). Tanycyte-like cells form a blood-cerebrospinal fluid barrier in the circumventricular organs of the mouse brain. J Comp Neurol.

[44] García M, Millán C, Balmaceda-Aguilera C, Castro T, Pastor P, Montecinos H, Reinicke K, Zúñiga F (2003). Hypothalamic ependymal-glial cells express the glucose transporter GLUT2, a protein involved in glucose sensing. J Neurochem.

[45] Millán C, Martínez F, Cortés-Campos C, Lizama I, Yañez MJ, Llanos P, Reinicke K, Rodríguez F (2010). Glial glucokinase expression in adult and post-natal development of the hypothalamic region. ASN Neuro.

[46] Cook AM, Mieure KD, Owen RD, Pesaturo AB, Hatton J (2009). Intracerebroventricular administration of drugs. Pharmacotherapy.

[47] Pardridge WM (2011). Drug transport in brain via the cerebrospinal fluid. Fluids Barriers CNS.

[48] Woods SC, McKay LD (1978). Intraventricular alloxan eliminates feeding elicited by 2-deoxyglucose. Science.

[49] Ritter S, Murnane JM, Ladenheim EE (1982). Glucoprivic feeding is impaired by lateral or fourth ventricular alloxan injection. Am J Physiol.

[50] Sanders NM, Dunn-Meynell AA, Levin BE (2004). Third ventricular alloxan reversibly impairs glucose counterregulatory responses. Diabetes.

[51] Lanfray D, Arthaud S, Ouellet J, Compère V, Do Rego JL, Leprince J, Lefranc B, Castel H (2013). Gliotransmission and brain glucose sensing: critical role of endozepines. Diabetes.

[52] Lacković Z, Salković M (1990). Streptozotocin and alloxan produce alterations in rat brain monoamines independently of pancreatic beta cells destruction. Life Sci.

[53] Sapcanin A, Sofic E, Tahirovic I, Salkovic-Petrisic M, Hoyer S, Riederer P (2008). Antioxidant capacity in rat brain after intracerebroventricular treatment with streptozotocin and alloxan—a preliminary study. Neurotox Res.

[54] Grünblatt E, Salkovic-Petrisic M, Osmanovic J, Riederer P, Hoyer S (2007). Brain insulin system dysfunction in streptozotocin intracerebroventricularly treated rats generates hyperphosphorylated tau protein. J Neurochem.

[55] Hoyer S, Müller D, Plaschke K (1994). Desensitization of brain insulin receptor. Effect on glucose/energy and related metabolism. J Neural Transm (Suppl).

[56] Salkovic-Petrisic M, Knezovic A, Hoyer S, Riederer P (2013). What have we learned from the streptozotocin-induced animal model of sporadic Alzheimer's disease, about the therapeutic strategies in Alzheimer's research. J Neural Transm.

[57] Hoyer S, Prem L, Sorbi S, Amaducci L (1993). Stimulation of glycolytic key enzymes in cerebral cortex by insulin. Neuroreport.

[58] Duelli R, Schröck H, Kuschinsky W, Hoyer S (1994). Intracerebroventricular injection of streptozotocin induces discrete local changes in cerebral glucose utilization in rats. Int J Dev Neurosci.

[59] Nitsch R, Hoyer S (1991). Local action of the diabetogenic drug, streptozotocin, on glucose and energy metabolism in rat brain cortex. Neurosci Lett.

[60] Hoyer S, Lee SK, Löffler T, Schliebs R (2000). Inhibition of the neuronal insulin receptor. An in vivo model for sporadic Alzheimer disease?. Ann N Y Acad Sci.

[61] Salkovic-Petrisic M, Osmanovic J, Grünblatt E, Riederer P, Hoyer S (2009). Modeling sporadic Alzheimer's disease: the insulin resistant brain state generates multiple long-term morphobiological abnormalities including hyperphosphorylated tau protein and amyloid-beta. J Alzheimers Dis.

[62] Correia SC, Santos RX, Perry G, Zhu X, Moreira PI, Smith MA (2011). Insulin-resistant brain state: the culprit in sporadic Alzheimer's disease?. Ageing Res Rev.

[63] Frisardi V, Solfrizzi V, Capurso C, Imbimbo BP, Vendemiale G, Seripa D, Pilotto A, Panza F (2010). Is insulin resistant brain state a central feature of the metabolic-cognitive syndrome?. J Alzheimers Dis.

[64] Anon (2003) American College of Endocrinology position statement on the insulin resistance syndrome. Endocr Pract 9:236-252 12924350

[65] Kaiyala KJ, Prigeon RL, Kahn SE, Woods SC, Schwartz MW (2000). Obesity induced by a high-fat diet is associated with reduced brain insulin transport in dogs. Diabetes.

[66] Salkovic-Petrisic M, Osmanovic-Barilar J, Brückner MK, Hoyer S, Arendt T, Riederer P (2011). Cerebral amyloid angiopathy in streptozotocin rat model of sporadic Alzheimer's disease: a long-term follow up study. J Neural Transm.

[67] Grieb P, Kryczka T, Fiedorowicz M, Frontczak-Baniewicz M, Walski M (2004). Expansion of the Golgi apparatus in rat cerebral cortex following intracerebroventricular injections of streptozotocin. Acta Neurobiol Exp (Wars).

[68] Labak M, Foniok T, Kirk D, Rushforth D, Tomanek B, Jasiński A, Grieb P (2010). Metabolic changes in rat brain following intracerebroventricular injections of streptozotocin: a model of sporadic Alzheimer's disease. Acta Neurochir Suppl.

[69] Correia SC, Santos RX, Santos MS, Casadesus G, Lamanna JC, Perry G, Smith MA, Moreira PI (2013). Mitochondrial abnormalities in a streptozotocin-induced rat model of sporadic Alzheimer's disease. Curr Alzheimer Res.

[70] Tiwari V, Kuhad A, Bishnoi M, Chopra K (2009). Chronic treatment with tocotrienol, an isoform of vitamin E, prevents intracerebroventricular streptozotocin-induced cognitive impairment and oxidative-nitrosative stress in rats. Pharmacol, Biochem Behav.

[71] Saxena G, Patro IK, Nath C (2011). ICV STZ induced impairment in memory and neuronal mitochondrial function: A protective role of nicotinic receptor. Behav Brain Res.

[72] Chen Y, Liang Z, Blanchard J, Dai CL, Sun S, Lee MH, Grundke-Iqbal I, Iqbal K (2013). A non-transgenic mouse model (icv-STZ mouse) of Alzheimer's disease: similarities to and differences from the transgenic model (3xTg-AD mouse). Mol Neurobiol.

[73] Rai S, Kamat PK, Nath C, Shukla R (2014). Glial activation and post-synaptic neurotoxicity: the key events in Streptozotocin (ICV) induced memory impairment in rats. Pharmacol, Biochem Behav.

[74] Santos TO, Mazucanti CH, Xavier GF, Torrão AS (2012). Early and late neurodegeneration and memory disruption after intracerebroventricular streptozotocin. Physiol Behav.

[75] Deng Y, Li B, Liu Y, Iqbal K, Grundke-Iqbal I, Gong CX (2009). Dysregulation of insulin signaling, glucose transporters, O-GlcNAcylation, and phosphorylation of tau and neurofilaments in the brain: Implication for Alzheimer's disease. Am J Pathol.

[76] Chen Y, Liang Z, Tian Z, Blanchard J, Dai CL, Chalbot S, Iqbal K, Liu F (2014). intracerebroventricular streptozotocin exacerbates Alzheimer-like changes of 3xTg-AD mice. Mol Neurobiol.

[77] de la Monte SM (2014). Type 3 diabetes is sporadic Alzheimer׳s disease: Mini-review. Eur Neuropsychopharmacol.

[78] de la Monte SM, Tong M (2014). Brain metabolic dysfunction at the core of Alzheimer's disease. Biochem Pharmacol.

[79] Lester-Coll N, Rivera EJ, Soscia SJ, Doiron K, Wands JR, de la Monte SM (2006). Intracerebral streptozotocin model of type 3 diabetes: relevance to sporadic Alzheimer's disease. J Alzheimers Dis.

[80] de la Monte SM, Tong M, Lester-Coll N, Plater M, Wands JR (2006). Therapeutic rescue of neurodegeneration in experimental type 3 diabetes: relevance to Alzheimer's disease. J Alzheimers Dis.

[81] Terwel D, Prickaerts J, Meng F, Jolles J (1995). Brain enzyme activities after intracerebroventricular injection of streptozotocin in rats receiving acetyl-L-carnitine. Eur J Pharmacol.

[82] Shoham S, Bejar C, Kovalev E, Weinstock M (2003). Intracerebroventricular injection of streptozotocin causes neurotoxicity to myelin that contributes to spatial memory deficits in rats. Exp Neurol.

[83] Kraska A, Santin MD, Dorieux O, Joseph-Mathurin N, Bourrin E, Petit F, Jan C, Chaigneau M (2012). In vivo cross-sectional characterization of cerebral alterations induced by intracerebroventricular administration of streptozotocin. PLoS One.

[84] Blondel O, Portha B (1989). Early appearance of in vivo insulin resistance in adult streptozotocin-injected rats. Diabete Metab.

[85] Kadowaki T, Kasuga M, Akanuma Y, Ezaki O, Takaku F (1984). Decreased autophosphorylation of the insulin receptor-kinase in streptozotocin-diabetic rats. J Biol Chem.

[86] Giorgino F, Chen JH, Smith RJ (1992). Changes in tyrosine phosphorylation of insulin receptors and a 170,000 molecular weight nonreceptor protein in vivo in skeletal muscle of streptozotocin-induced diabetic rats: effects of insulin and glucose. Endocrinology.

[87] Reed BC, Lane MD (1980). Insulin receptor synthesis and turnover in differentiating 3 T3-L1 preadipocytes. Proc Natl Acad Sci U S A.

[88] Schubert M, Gautam D, Surjo D, Ueki K, Baudler S, Schubert D, Kondo T, Alber J (2004). Role for neuronal insulin resistance in neurodegenerative diseases. Proc Natl Acad Sci U S A.

[89] Iliff JJ, Wang M, Liao Y, Plogg BA, Peng W, Gundersen GA, Benveniste H, Vates GE (2012). A paravascular pathway facilitates CSF flow through the brain parenchyma and the clearance of interstitial solutes, including amyloid β. Sci Transl Med.

[90] Havrankova J, Roth J, Brownstein MJ (1979). Concentrations of insulin and insulin receptors in the brain are independent of peripheral insulin levels. Studies of obese and streptozotocin-treated rodents. J Clin Invest.

[91] Schechter R, Whitmire J, Holtzclaw L, George M, Harlow R, Devaskar SU (1992). Developmental regulation of insulin in the mammalian central nervous system. Brain Res.

[92] Banks WA, Owen JB, Erickson MA (2012). Insulin in the brain: there and back again. Pharmacol Ther.

[93] Schechter R, Beju D, Miller KE (2005). The effect of insulin deficiency on tau and neurofilament in the insulin knockout mouse. Biochem Biophys Res Commun.

[94] Salkovic-Petrisic M, Tribl F, Schmidt M, Hoyer S, Riederer P (2006). Alzheimer-like changes in protein kinase B and glycogen synthase kinase-3 in rat frontal cortex and hippocampus after damage to the insulin signalling pathway. J Neurochem.

[95] Salkovic-Petrisic M, Riederer P (2010). Brain glucose transporter protein 2 and sporadic Alzheimer's disease. Transl Neurosci.

[96] Arluison M, Quignon M, Nguyen P, Thorens B, Leloup C, Penicaud L (2004). Distribution and anatomical localization of the glucose transporter 2 (GLUT2) in the adult rat brain—an immunohistochemical study. J Chem Neuroanat.

[97] Rodríguez EM, Blázquez JL, Pastor FE, Peláez B, Peña P, Peruzzo B, Amat P (2005). Hypothalamic tanycytes: a key component of brain-endocrine interaction. Int Rev Cytol.

[98] Coimbra CC, Migliorini RH (1986). Insulin-sensitive glucoreceptors in rat preoptic area that regulate FFA mobilization. Am J Physiol.

[99] Kraegen EW, Cooney GJ (2008). Free fatty acids and skeletal muscle insulin resistance. Curr Opin Lipidol.

[100] Smith QR, Nagura H (2001). Fatty acid uptake and incorporation in brain: studies with the perfusion model. J Mol Neurosci.

[101] Patil S, Melrose J, Chan C (2007). Involvement of astroglial ceramide in palmitic acid-induced Alzheimer-like changes in primary neurons. Eur J Neurosci.

[102] Liu L, Martin R, Kohler G, Chan C (2013). Palmitate induces transcriptional regulation of BACE1 and presenilin by STAT3 in neurons mediated by astrocytes. Exp Neurol.

[103] de la Monte SM (2012). Metabolic derangements mediate cognitive impairment and Alzheimer's disease: role of peripheral insulin-resistance diseases. Panminerva Med.

[104] Tong M, Neusner A, Longato L, Lawton M, Wands JR, de la Monte SM (2009). Nitrosamine exposure causes insulin resistance diseases: relevance to type 2 diabetes mellitus, non-alcoholic steatohepatitis, and Alzheimer's disease. J Alzheimers Dis.

